# GABA_B_-Receptor Agonist-Based Immunotherapy for Type 1 Diabetes in NOD Mice

**DOI:** 10.3390/biomedicines9010043

**Published:** 2021-01-06

**Authors:** Jide Tian, Blake Middleton, Victoria Seunghee Lee, Hye Won Park, Zhixuan Zhang, Bokyoung Kim, Catherine Lowe, Nancy Nguyen, Haoyuan Liu, Ryan S. Beyer, Hannah W. Chao, Ryan Chen, Davis Mai, Karen Anne O’Laco, Min Song, Daniel L. Kaufman

**Affiliations:** Department of Molecular and Medical Pharmacology, University of California, Los Angeles, CA 90095-1735, USA; bmiddleton@mednet.ucla.edu (B.M.); victoriacl14@gmail.com (V.S.L.); hyewonpark26@gmail.com (H.W.P.); susanzz07@g.ucla.edu (Z.Z.); bkkatekim1@gmail.com (B.K.); cal2@me.com (C.L.); nancy.nguyen1024@gmail.com (N.N.); liuhaoy67@gmail.com (H.L.); ryan.s.beyer@gmail.com (R.S.B.); hannah.chao2@gmail.com (H.W.C.); rchen818@ucla.edu (R.C.); davistmai@gmail.com (D.M.); KOlaco@mednet.ucla.edu (K.A.O.); minsong@mednet.ucla.edu (M.S.)

**Keywords:** GABA receptor, type 1 diabetes, autoimmune disease, immunotherapy, GABA_B_-R, GABA_A_-R

## Abstract

Some immune system cells express type A and/or type B γ-aminobutyric acid receptors (GABA_A_-Rs and/or GABA_B_-Rs). Treatment with GABA, which activates both GABA_A_-Rs and GABA_B_-Rs), and/or a GABA_A_-R-specific agonist inhibits disease progression in mouse models of type 1 diabetes (T1D), multiple sclerosis, rheumatoid arthritis, and COVID-19. Little is known about the clinical potential of specifically modulating GABA_B_-Rs. Here, we tested lesogaberan, a peripherally restricted GABA_B_-R agonist, as an interventive therapy in diabetic NOD mice. Lesogaberan treatment temporarily restored normoglycemia in most newly diabetic NOD mice. Combined treatment with a suboptimal dose of lesogaberan and proinsulin/alum immunization in newly diabetic NOD mice or a low-dose anti-CD3 in severely hyperglycemic NOD mice greatly increased T1D remission rates relative to each monotherapy. Mice receiving combined lesogaberan and anti-CD3 displayed improved glucose tolerance and, unlike mice that received anti-CD3 alone, had some islets with many insulin^+^ cells, suggesting that lesogaberan helped to rapidly inhibit β-cell destruction. Hence, GABA_B_-R-specific agonists may provide adjunct therapies for T1D. Finally, the analysis of microarray and RNA-Seq databases suggested that the expression of GABA_B_-Rs and GABA_A_-Rs, as well as GABA production/secretion-related genes, may be a more common feature of immune cells than currently recognized.

## 1. Introduction

Gamma-aminobutyric acid receptors (GABA-Rs) have been extensively studied in the central nervous system (CNS) for their roles in modulating neuronal activity. There is a growing body of evidence that immune cells also express GABA-Rs and that the activation of these receptors generally has anti-inflammatory effects. There are two types of GABA-Rs that are encoded by different gene families and act through different intracellular pathways. GABA_A_-Rs are fast-acting chloride channels that are pentameric hetero- or homomeric receptors formed from 19 GABA_A_-R subunits (α_1–6_, β_1–3_, γ_1–3_, δ, ε, π, θ, and ρ_1–3_) [1]. In contrast, GABA_B_-Rs are slow-acting G-protein-coupled receptors formed by heterodimers of the products of the *GABBR1* and *GABBR2* genes [2].

Pharmacological and electrophysiological studies have functionally demonstrated that human and murine T cells express GABA_A_-Rs [3,4,5,6,7,8,9,10,11]. Oral treatment with GABA, which activates both GABA_A_-Rs and GABA_B_-Rs, or the GABA_A_-R-specific agonist homotaurine, reduces autoantigen-specific Th1 and Th17 responses, enhances CD4^+^ and CD8^+^ Treg responses, downregulates antigen-presenting cell (APC) pro-inflammatory activities, and ameliorates disease in mouse models of T1D, rheumatoid arthritis, or experimental autoimmune encephalomyelitis [7,12,13,14,15,16]. Our recent studies indicate that GABA treatment can also limit pulmonary inflammation in a mouse model of COVID-19 and thereby greatly reduce disease severity and death rates [17].

Much less is known about the roles that GABA_B_-Rs play in immunobiology. Studies using the clinically applicable GABA_B_-R-specific agonist baclofen have observed that baclofen treatment can: (1) inhibit murine dendritic cell (DC) activation and immune cell chemotaxis [18,19], (2) inhibit DC proinflammatory functions [18], (3) alleviate collagen-induced arthritis and contact dermatitis in mouse models [18,19], (4) delay T1D onset in NOD mice [20], and (5) attenuate TLR4-induced inflammatory signaling in human peripheral blood mononuclear cells (PBMCs) [21]. Baclofen, as well as most other clinically applicable GABA_B_-R specific agonists, however, are ill-suited for the treatment of inflammatory disorders in the periphery since these drugs cross the blood–brain barrier and modulate neuronal activity in the CNS. Therefore, the development or repurposing of GABA_B_-R agonists that are peripherally restricted and safe for human use may have clinical potential for reducing inflammation while avoiding CNS side effects.

The insulin-producing β-cells in pancreatic islets also express GABA_A_-Rs and GABA_B_-Rs [22,23,24,25,26,27]. The activation of either of these receptors has been shown to promote β-cell survival and replication in streptozotocin-rendered diabetic mice and human islets implanted into immune-deficient mice [5,14,16,22,24,26,27,28,29,30,31]. Accordingly, treatment with GABA_B_-R agonists may have multiple beneficial effects for T1D treatment due to their actions on both β-cells and immune cells.

Lesogaberan (AZD3355) is a peripherally restricted GABA_B_-R agonist that was developed for the treatment of gastroesophageal reflux disease (GERD) [32,33,34]. It has an EC_50_ of 9 nM (compared to GABA’s EC_50_ of 160 nM), a K_i_ of 5 nM (vs. 110 nM for GABA) for GABA_B_-Rs [32], and a half-life of about 11 h in peripheral blood [35,36]. While treatment of GERD patients with lesogaberan in several phase IIb clinical trials did not lead to sufficient beneficial effects, there were no treatment-related serious adverse events [33,35,37]. Our previous study showed that similar to GABA, lesogaberan enhances the replication of human islet cells through the activation of islet GABA_B_-Rs in vitro and oral lesogaberan treatment promotes the survival and replication of human β-cells in NOD/SCID mice [38].

Here, we investigated lesogaberan’s potential as an interventive therapy in diabetic NOD mice. A large number of treatments have been shown to prevent or delay the onset of T1D in prediabetic NOD mice (reviewed in [39]); however, few have displayed an ability to correct hyperglycemia after disease onset in NOD mice, which is the clinically relevant situation. We tested whether lesogaberan alone or in combination with an antigen-specific immunotherapy (proinsulin/alum immunization) or an immunodepletive therapy (anti-CD3) could modulate the progression of T1D in newly and severely diabetic NOD mice. Finally, our analysis of published microarray and RNA-Seq gene expression profiles suggests that most murine and human immune cells express the components needed to form GABA_B_-Rs and GABA_A_-Rs, as well as to produce, secrete and take up GABA.

## 2. Materials and Methods

### 2.1. Reagents

Lesogaberan was supplied by AstraZeneca (London, UK). The development and structure of lesogaberan have been previously described [34]. GABA was purchased from Sigma-Aldrich (St. Louis, MO, USA). Hamster anti-CD3ε 2C11 F(ab′)2 fragment (anti-CD3) was obtained from BioXCell (West Lebanon, NH, USA).

### 2.2. Mice

NOD mice were originally from Taconic Biosciences (Germantown, NY, USA) and maintained in our specific pathogen-free facility. Female NOD mice were used in this study, because they develop a high frequency of T1D. This study was carried out in accordance with the recommendations of the Guide for the Care and Use of Laboratory Animals of the National Institutes of Health. The protocols for all experiments using vertebrate animals were approved by the Animal Research Committee at UCLA (Protocol ID: ARC # 1993-211; Date: 4/14/17–4/13/2023).

### 2.3. Lesogaberan Monotherapy

Female NOD mice with blood glucose levels between 250 and 300 mg/dL for two consecutive days were recruited into the study. The mice were randomized and treated with plain drinking water or drinking water containing 0.025, 0.08, 0.25, or 0.75 mg/mL of lesogaberan. Fresh lesogaberan-containing water was prepared each week. The animals’ blood glucose levels were monitored for up to 28 weeks post-T1D onset. Mice with two consecutive blood glucose readings of <250 mg/dL were considered to be in remission, after which two consecutive blood glucose readings of >250 mg/dL were considered as disease relapse.

### 2.4. Combined Lesogaberan and Antigen-Specific Immunotherapy Treatment

Newly diabetic mice (two consecutive blood glucose levels of 250–300 mg/dL) were treated with proinsulin (100 µg, kindly provided by Eli Lilly) in 50% alum intraperitoneally. The same day, the animals were placed on water containing a suboptimal dose of lesogaberan (0.08 mg/mL), which was continued until indicated. The mice were boosted with proinsulin/alum 10 days later and were monitored for disease remission and relapse as described above.

### 2.5. Combined Lesogaberan and Low-Dose Anti-CD3 Treatment in Severely Diabetic NOD Mice

To assess whether lesogaberan could augment the efficacy of an effector T cell-depletive therapy after the establishment of severe hyperglycemia, we treated diabetic NOD mice that had a blood glucose reading of >340 mg/dL with low-dose anti-CD3 (35 µg) for three consecutive days, a treatment which was observed to be partially effective in previous studies [16]. Mice with two consecutive blood glucose readings between 340 and 550 mg/dL within 1 week of the initial treatment were included in our subsequent analysis. At the time of the first anti-CD3 treatment, the animals were randomized to receive plain water or water containing lesogaberan (0.08 mg/mL, continuously) and monitored for disease remission and relapse for up to 25 weeks. At 25 weeks post-initiating treatment, the pancreas from some surviving mice were processed for immunofluorescent staining with anti-insulin, anti-glucagon, and DAPI, as previously described [40]. Some mice that had been treated with combined therapies were provided with plain water alone (withdrawal of lesogaberan) at 25 weeks post-treatment, and they were monitored for T1D relapse for another 25 weeks.

### 2.6. Intraperitoneal Glucose Tolerance (IPGT) Test

Twenty-five weeks after initiating treatment, some mice were fasted for 16 h, and their blood glucose levels were monitored just before, as well as 15, 30, 60, 90, 120, and 180 min post-challenge with glucose (2 g/kg IP). The areas under the curve of the blood glucose levels were calculated.

## 3. Results

### 3.1. Lesogaberan Treatment Can Temporarily Correct Hyperglycemia in Newly Diabetic NOD Mice

**Figure 1:**
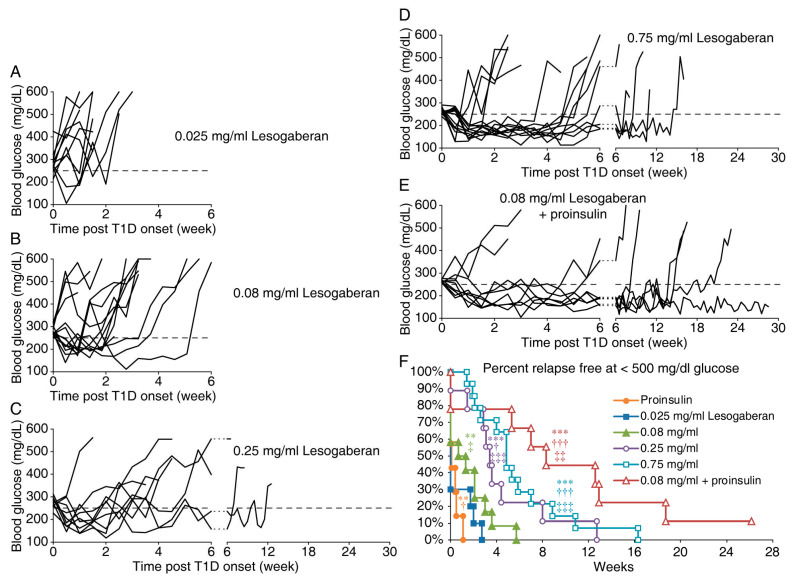
Longitudinal blood glucose levels in newly diabetic NOD mice given lesogaberan over a dose range or combined treatment with proinsulin/alum immunization. Newly diabetic NOD mice were treated with lesogaberan at 0.025 mg/mL (*n* = 10) (**A**), 0.08 mg/mL (*n* = 12) (**B**), 0.25 mg/mL (*n* = 9) (**C**), or 0.75 mg/mL (*n* = 14) (**D**). Data shown are longitudinal blood glucose levels for individual mice. The dashed line indicates a blood glucose of 250 mg/dL. (**E**) In a subsequent study, we treated newly diabetic NOD mice with a suboptimal dose of lesogaberan (0.08 mg/mL) together with proinsulin/alum immunization (*n* = 9). (**F**) Data show the percent of relapse-fee mice in each treatment group over the time of post-treatment. Data for proinsulin/alum monotherapy were previously reported [40] and were reproduced here for reference. ** *p* < 0.01 and *** *p* < 0.01 vs. the controls with plain water; ^†^ *p* < 0.05, and ^†††^ *p* < 0.001 vs. the mice with 0.08 mg/mL lesogaberan (alone); and ^‡^ *p* < 0.05, ^‡‡^ *p* < 0.01, and ^‡‡‡^ *p* < 0.001 vs. the mice with proinsulin alone as determined by the log-rank test.

To determine whether lesogaberan had therapeutic potential for the treatment of T1D, newly diabetic NOD mice (two consecutive blood glucose levels between 250 and 350 mg/dL) were randomized and placed on plain water (controls) or water containing 0.025, 0.08, 0.25, or 0.75 mg/mL of lesogaberan. All mice that received plain water rapidly progressed to severe hyperglycemia within one week. Mice that received the lowest dose of lesogaberan (0.025 mg/mL) did not significantly respond to therapy (Figure 1A, *p* = 0.07 vs. the plain water-treated). About half of the mice that received lesogaberan at 0.08 mg/mL displayed a very brief remission that lasted a mean of 1.5 weeks (Figure 1B, *p* < 0.005 vs. the control). Most of the mice that received a higher dosage of 0.25 or 0.75 mg/mL lesogaberan went into remission, which lasted a mean of 4.4 and 5.8 weeks, respectively (Figure 1C,D, both *p* < 0.001 vs. the control). Lesogaberan at 0.25 and 0.75 mg/mL were significantly more effective than the 0.08 mg/mL dose (*p* < 0.05 and <0.001, respectively), but the difference in the therapeutic effects between 0.25 and 0.75 mg/mL of lesogaberan was not significant (*p* = 0.3). Thus, lesogaberan displayed a dose-dependent ability to temporarily correct hyperglycemia in newly diabetic NOD mice.

### 3.2. The Combination of a Low Dose of Lesogaberan and Proinsulin/Alum Treatment Prolongs the Disease Remission Periods in Newly Diabetic NOD Mice

We next asked whether the combination treatment with lesogaberan and an antigen-specific therapy, proinsulin/alum immunization, could enhance therapeutic benefit. We chose to test the lowest dose of lesogaberan (0.08 mg/mL) that significantly induced diabetes remission, although mice receiving that dose displayed an average time of remission of only 1.5 weeks. We previously reported that proinsulin/alum immunization had little ability to induce diabetes remission in newly diabetic NOD mice [40] and that data were reproduced in Figure 1F. Here, we observed that 2/3 of the newly diabetic mice receiving the combined therapy became normoglycemic and remained normoglycemic for at least 5 weeks. The mice receiving combined treatments had a mean remission period of 10.1 weeks (Figure 1E), which was a statistically significant increase compared to those receiveing either monotherapy (*p* = 0.001 and 0.003 for lesogaberan and proinsulin monotherapy, respectively). The percentages of relapse-free mice in all groups are shown in Figure 1F. Thus, the combination of a low dose of lesogaberan and proinsulin/alum treatments greatly prolonged the diabetes remission periods in newly diabetic NOD mice.

### 3.3. Combination of a Low Dose of Lesogaberan and Anti-CD3 Treatments Synergistically Increases the Frequency of Severely Diabetic NOD Mice with Diabetes Remission

**Figure 2:**
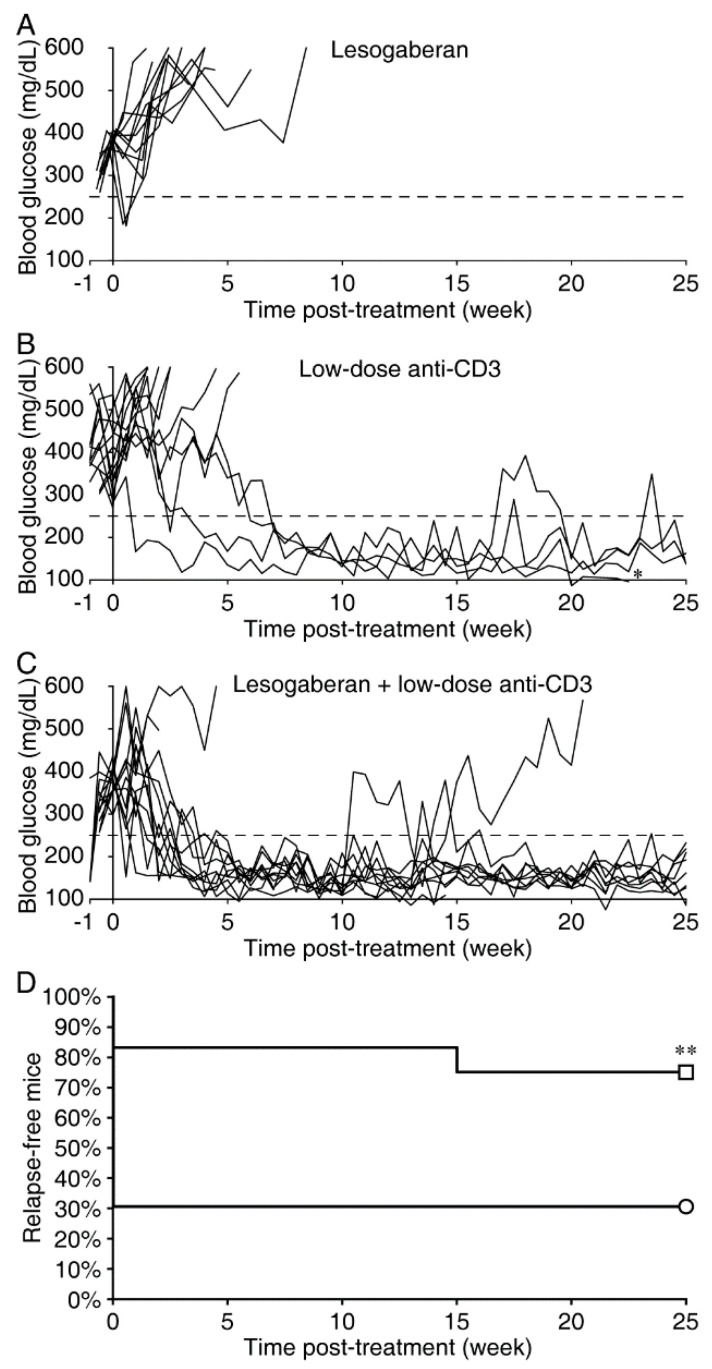
Combined low-dose lesogaberan and anti-CD3 treatment increases the long-term disease remission rates in severely diabetic NOD mice. Longitudinal blood glucose readings of individual severely diabetic NOD mice (blood glucose of 340–550 mg/dL) that were given lesogaberan (0.08 mg/mL) (*n* = 11) (**A**), low-dose anti-CD3 (*n* = 13) (**B**), or combined low-dose anti-CD3 and lesogaberan (0.08 mg/mL) (*n* = 12) (**C**). * Died of unknown causes. (**D**) Data show the percents of relapse-fee mice in groups of mice given anti-CD3 (⚪) or the combination of anti-CD3 and lesogaberan (□) for 25-weeks following the treatment. **p = 0.01 as determined by the log-rank test. These studies were run concurrently with studies of combined homotaurine and low-dose anti-CD3 treatment, and the control anti-CD3 group data were previously presented in [16].

Anti-CD3 treatment has shown promise to correct hyperglycemia in individuals newly diagnosed with T1D and to delay disease onset in those at high risk for T1D [41]. Since individuals are generally severely hyperglycemic at the time they are diagnosed with T1D, we tested the therapeutic effect of lesogaberan and anti-CD3 on severely diabetic NOD mice (two consecutive blood glucose readings between 340 and 550 mg/dL). Because there are concerns about possible side effects of immunodepletive therapies, we previously developed a low-dose anti-CD3 protocol, which induced diabetes remission in about one-third of all treated severely diabetic NOD mice [16]. We treated the severely diabetic NOD mice with low-dose anti-CD3 and placed them on plain water or a suboptimal dose of lesogaberan (0.08 mg/mL). Another group of mice received lesogaberan alone (0.08 mg/mL). Lesogaberan monotherapy had no ability to slow disease progression in these mice (Figure 2A). Low-dose anti-CD3 treatment led to disease remission in 31% of the treated animals with half of these responder mice taking over 5 weeks to become normoglycemic (Figure 2B,D). Impressively, the combination of a low dose of anti-CD3 and lesogaberan increased the remission rate to 83% of mice (*p* = 0.01 vs. the mice with low-dose anti-CD3 monotherapy), and all of these mice went into remission in <4 weeks post-treatment (Figure 2C). Ninety percent of the mice that responded to the combined therapy remained in remission throughout the 25-week observation period (Figure 2C,D). Thus, the combination of a low dose of lesogaberan and anti-CD3 significantly increased the remission rates in severely diabetic NOD mice.

After 25 weeks of giving anti-CD3-treated mice oral lesogaberan, some mouse pancreata were examined histologically as described below. We withdrew the oral lesogaberan treatment from the remaining mice and followed them for 48–50 weeks post-initiation of treatment. All of these mice remained normoglycemic over that extended observation period.

### 3.4. Functional and Histological Assessments of Islets in Mice Given Low Doses of Anti-CD3 and Lesogaberan

**Figure 3:**
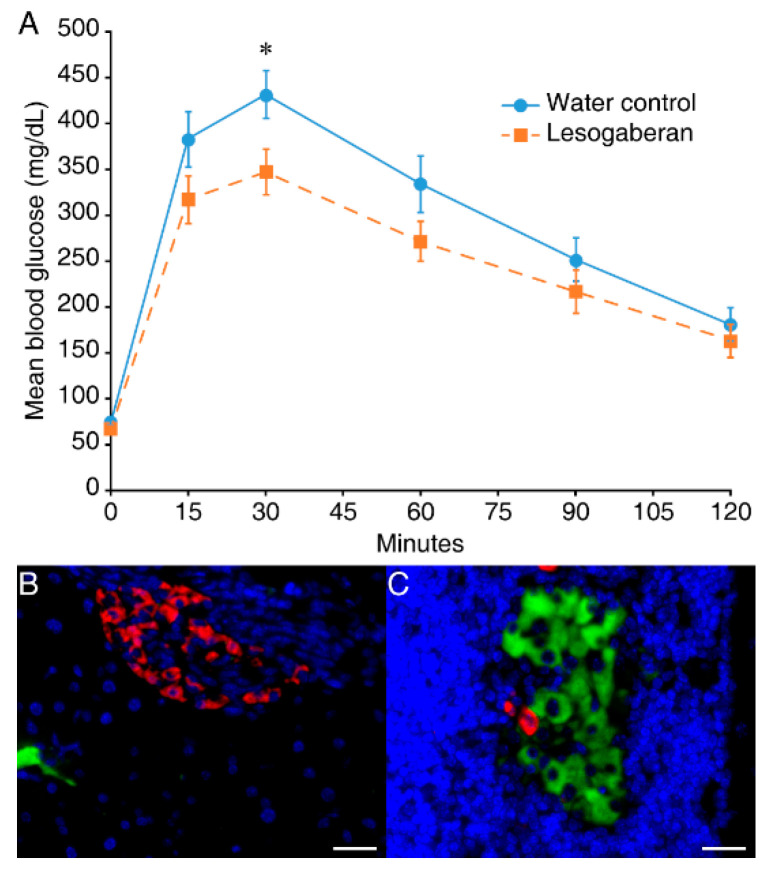
Combined therapy improves glucose tolerance and preserves some functional islets. (**A**) Mice that remained in remission for 25 weeks post-anti-CD3 monotherapy or combined anti-CD3/ lesogaberan therapies were given an IPGT test. Data shown are mean glucose (mg/dL) over time (minutes) ± SEM of the groups of mice given anti-CD3 monotherapy (solid line and circle symbols) or combined anti-CD3/lesogaberan therapies (dashed line with square symbols). * *p* = 0.031 for combined therapy versus anti-CD3 monotherapy at 30 min by repeated measures ANOVA. *n* = 10 mice per group. (**B**) A representative islet image with few insulin^+^ cells in the mice receiving anti-CD3 (alone) that was co-stained with anti-insulin (green), anti-glucagon (red), and DAPI. (**C**) A representative islet image with many insulin^+^ cells in the mice receiving combined therapies. Scale bar: 25 µm.

We assessed glucose tolerance in the mice that remained in remission 25 weeks after the anti-CD3 (alone) and combined anti-CD3/lesogaberan therapy. Although mice in both of these treatment groups were normoglycemic, when given a glucose challenge, the mice that received combined therapy displayed significantly better glucose tolerance than those which received anti-CD3 monotherapy (Figure 3A).

The immunofluorescent analysis revealed that there were few insulin^+^ cells in the islets from the NOD mice that responded to low-dose anti-CD3 (alone) when examined 25 weeks post-treatment and these islets were insulitis-free (a representative image is shown in Figure 3B). In contrast, in the mice receiving combined therapies, there were a few islets with many insulin^+^ cells that were surrounded by mononuclear infiltrates (Figure 3C). Therefore, the combination of a low dose of anti-CD3 and lesogaberan treatments preserved functional islets in severely diabetic NOD mice.

## 4. Discussion

Previous studies have suggested that GABA_B_-R agonists might be useful for helping to treat T1D since β-cells express GABA_B_-Rs and their activation promotes β-cell replication and survival in STZ-rendered diabetic mice and human islet xenografts implanted into immunodeficient mice [28,29]. However, this capability may not have clinical utility if autoimmune responses to β-cells cannot be controlled. In that regard, previous studies have shown that the GABA_B_-R agonist baclofen can inhibit APC activation and functions [18,19,20,21,42] and thereby indirectly limit Th17 and Th1 responses, which may have contributed to the observation that baclofen treatment delays the onset of T1D in prediabetic NOD mice [20].

In our studies of newly diabetic NOD mice, lesogaberan treatment had a modest, dose-dependent ability to correct hyperglycemia. The combination of a suboptimal dose of lesogaberan and proinsulin/alum immunization greatly increased the frequency of diabetic mice undergoing remission and significantly prolonged the disease remission periods. Additionally, the combination treatment with low doses of lesogaberan and anti-CD3 further increased the rate of disease remission for at least 25 weeks in the severely diabetic NOD mice. The lesogaberan treatment was subsequently withdrawn, and all of the mice remained normoglycemic up to the end of another 25-week observation period.

Our assessment of glucose tolerance in the mice that remained in remission 25 weeks after anti-CD3 (alone) and combined anti-CD3/lesogaberan therapy revealed that while these treatments restored normoglycemia, the mice that received combined therapy had significantly better glucose tolerance than those which received anti-CD3 alone. The histological analysis revealed that most mice given combined anti-CD3 and lesogaberan treatments, but not those receiving anti-CD3 alone, had a few islets with many insulin^+^ β-cells. These functional islets had a surrounding peri-insulitis, as has been observed in past studies of anti-CD3-treated diabetic mice which was reported to be primarily comprised of CD4^+^ and CD8^+^ T cells, B cells, and macrophages [43,44,45,46,47]. We believe that these few functional islets reflect the ability of lesogaberan to (1) rapidly inhibit proinflammatory APC activities and consequently limit pathogenic T cell activities and (2) activate β-cell GABA_B_-Rs to limit β-cell apoptosis, thereby helping to preserve the few remaining functional islets at the time when combined treatment was initiated. These data suggest that short-term treatment with a low-dose immunodepletive agent and a GABA_B_-R agonist may establish durable disease remission in diabetic individuals.

In severely hyperglycemic mice, it was notable that combined lesogaberan and anti-CD3 acted more quickly than anti-CD3 alone to correct hyperglycemia, with responders becoming normoglycemic within 2–3 weeks following the treatment. This rapid therapeutic effect is unlikely to arise from lesogaberan’s ability to enhance β-cell replication. It is possible that lesogaberan helped to correct hyperglycemia by acting on islet β-cell or α-cell GABA_B_-Rs and modulating insulin and/or glucagon release. However, the GABA_B_-R agonist baclofen is known to impair glucose intolerance and diminish insulin secretion in mice which is counter to our observations that lesogaberan treatment promoted normoglycemia [48]. We cannot rule out the possibility that by diminishing insulin secretion, lesogaberan reduced β-cell stress and thereby helped preserve some β-cells. Given the ability of GABA_B_-R agonists to limit APC proinflammatory activities [18,19], we favor the notion that lesogaberan, together with the low-dose anti-CD3, rapidly curtails autoimmune-mediated destruction of β-cells, consistent with the observed preservation of some functional islets in the mice that received combined therapy.

We previously studied the effects of homotaurine, a GABA_A_-R-specific agonist, in combination with proinsulin/alum and low-dose anti-CD3 [16]. Although homotaurine activates GABA_A_-Rs and lesogaberan activates GABA_B_-Rs, both compounds had similar abilities to restore euglycemia and preserve islet β-cell function. Since the current study used lesogaberan at a suboptimal dose in these combination therapies, it is difficult to draw more extensive conclusions from comparing these two studies.

Although GABA_A_-R agonists have been observed to induce Tregs in vivo [14,15,16,49], there is no current evidence of GABA_B_-R agonists directly modulating T cells. We cannot exclude the possibility that the GABA_B_-R agonist lesogaberan may have indirectly altered the frequency and function of Tregs by modulating APCs or modulating the gut microbiota. However, in our studies of murine experimental autoimmune encephalomyelitis (EAE), we have observed that a blood–brain barrier-permeable GABA_A_-R-specific agonist (homotaurine), but not GABA which activates both GABA_A_-Rs and GABA_B_-Rs, was capable of modulating the disease course, suggesting that GABA_B_-Rs in the gut or GABA-mediated changes in the gut microbiome did not play a discernable role in modulating the course of EAE (Tian et al. submitted).

Clinical trials with lesogaberan for GERD administered oral doses of 120–480 mg per day [33,35,37,50,51]. In our mouse studies, we administered lesogaberan through drinking water at 0.08 mg/mL as a monotherapy and in combination with proinsulin/alum or anti-CD3. The human equivalent dose (based on the mice drinking 4 mL/day) would be about 65 mg/day for a 70 kg individual [52], well below the dose used in previous clinical trials. While lesogaberan at 0.08 mg/mL did not display significant efficacy as a monotherapy in severely diabetic NOD mice, it enhanced the therapeutic efficacy of low-dose anti-CD3 in severely diabetic NOD mice. Thus, in the combination treatment, a subclinical dose of lesogaberan was therapeutically effective. Lesogaberan at 0.25 mg/mL was efficacious as a monotherapy (and to a similar extent as the 0.75 mg/mL dose) and corresponded to a human equivalent dose of about 202 mg/day, which is within the range of the doses used in human clinical trials. Thus, these preclinical findings suggested that lesogaberan may provide an adjunct therapy for a range of peripheral inflammatory disorders at dosages below or similar to those used in previous clinical trials for GERD. Since inflammation plays a major role in T2D and GABA_B_-R agonists can promote human β-cell replication and function [28,29], lesogaberan may also have the potential to help ameliorate T2D.

The types of immune cells that express GABA_B_-Rs and their immunobiological functions have not been systematically studied. Past reports have indicated that activation of GABA_B_-Rs can directly and/or indirectly modulate the activities of cells of the innate immune system. The pharmacological or genetic blockage of GABA_B_-Rs in murine DCs inhibited their production of IL-6 and the priming of Th17 cells, and baclofen treatment alleviated murine collagen-induced arthritis [18]. Additionally, baclofen reduced human PBMC chemotaxis towards chemokines, reduced PHA-stimulated human PBMC secretion of TNFα and ameliorated contact dermatitis in mice [19]. Baclofen also attenuated human PBMC TLR4-induced signaling [21] and stimulated human neutrophil chemotaxis [53]. Finally, GABA_A_-R antagonists (primarily), but also GABA_B_-R antagonists, attenuated the hypermigration of parasite-infected human DCs [42].

To further assess whether GABA_B_-Rs may modulate the functions of different immune cell types, we examined the microarray gene expression profiles of different types of murine immune cells in the Immgen database (http://www.immgen.org/Databrowser19/DatabrowserPage.html). GABA_B_-Rs are heterodimers of *GABBR1* and *GABBR2* gene products and engineered cell lines or transgenic mice that express only one of these genes lack functional GABA_B_-Rs [54,55,56,57]. The inspection of the *GABBR1* and *GABBR2* gene expression profiles of various types of DCs, NK cells, and monocytes provided clear evidence of *GABBR1* and *GABBR2* expression which could enable the formation of GABA_B_-Rs. Murine T cells also expressed *GABBR1* and *GABBR2* at levels similar to those detected in the aforementioned APC, which is unexpected, since pharmacological studies have observed that murine and human T cell function is modulated by GABA_A_-R, but not GABA_B_-R, agonists and antagonists (see discussions below).

**Figure 4:**
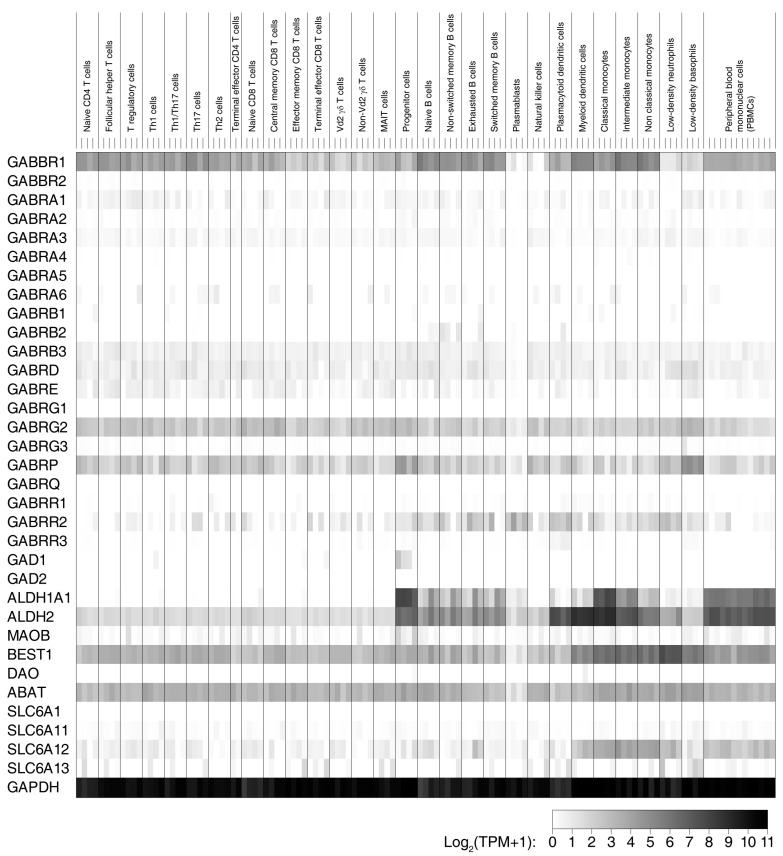
Heat map representation of the expression of genes related to the GABA system in 29 different human immune cell types as determined by single-cell RNA-Seq. The transcript expression profiles in 29 human immune cell types isolated from four individuals as well as in the peripheral blood mononuclear cells (PBMCs) from 13 individuals were obtained by Monaco et al. [58]. The relative levels of transcripts encoding GABA_B_-R subunits (GABBR1 and GABBR2), GABA_A_-R subunits (GABRA1–6, GABRB1–3, GABRD, GABRE, GABRG1–3, GABRP, and GABRR1–3), GABA synthetic enzymes GAD67 and GAD65 (GAD1 and GAD2, respectively), GABA synthesis and secretion via the putrescine pathway (ALDH1A1, MAOB, BEST1, or DAO), GABA degradation (ABAT), GABA transporters (SLC6A1, SLC6A11, SLC6A12, and SLC6A13), and the “housekeeping” gene glyceraldehyde 3-phosphate dehydrogenase (GAPDH) are shown. Transcript expression values in transcripts per million (TPM) for these genes were taken from Monaco et al. [58], log2 transformed with a pseudocount of 1 and plotted as a heat map. The raw numerical data are shown in Supplemental Table S1.

We also assessed the expression genes involved in the GABA signaling system by analyzing the RNA-Seq data sets from 29 subsets of human immune cells that were obtained by Monaco et al. [58]. The expression profiles of genes involved in the formation of GABA_B_-Rs and GABA_A_-Rs as well as the synthesis of GABA through the glutamic acid decarboxylase (GAD) pathway (via GAD65 and/or GAD67 [59]) or via the putrescine and/or the diamine oxidase pathways [60,61,62,63] and those involved in the GABA uptake and degradation are shown in Figure 4 and Supplemental Table S1.

In contrast to murine immune cells, the expression patterns of *GABBR1* and *GABBR2* in human APC and T cells as determined by RNA-Seq were highly skewed, with relatively high expression of *GABBR1* (particularly in B cells, DCs, and monocytes), but little or no detectable *GABBR2* expression (Figure 4 and Supplementary Table S1). Consistent with these RNA-Seq data, *GABBR1*, but not *GABBR2*, was detected in human PBMCs by RT-qPCR [64]. To reconcile these findings with the functional evidence of GABA_B_-R-mediated modulation of human APCs noted above, it is possible that low levels of GABBR2 are sufficient to form GABA_B_-Rs that modulate APC functions or that the GABBR1 subunit on its own has some intracellular activity as previously reported [65,66,67]. Alternatively, some of the observations of GABA_B_-R agonist’s immunological effects may be due to indirect or off-target effects. If human APCs have functional GABA_B_-Rs, it is possible that various subsets of human T cells (including Th1, Th17, Tregs, and CD8^+^ T cells) and B cells also possess GABA_B_-Rs since their expression levels of *GABBR1* and *GABBR2* are similar to those of various human APCs (Figure 4, Supplementary Table S1). Since there is strong pharmacological and electrophysiological evidence that GABA’s anti-inflammatory effects on mouse and human T cells are mediated through GABA_A_-Rs [3,5,8,9,15,16], if T cells do possess functional GABA_B_-Rs their activation may have a little impact on the anti-inflammatory effects of GABA_A_-R activation.

The analysis of GABA_A_-R subunit expression in human NK cells, DCs, and monocytes from different individuals revealed the prominent expression of α3, along with generally lower levels or less frequent detection of α1, α2, α4, and α6 (Figure 4 and Supplemental Table S1). The inclusion of α3, α1, or α2 in GABA_A_-Rs confers sensitivity to benzodiazepines [68]. These cells also consistently expressed ß3 and δ subunits, along with relatively high levels of γ2 and π subunits and lower levels or less frequent detection of the ε and γ3 subunits. Accordingly, these cells expressed the required components to form heteropentameric GABA_A_-Rs. In addition, these cells prominently expressed the ρ_2_ subunit which is capable of forming GABA_A_-Rs homo-oligomers [69,70]. A similar expression profile was found in different subsets of T cells (naïve CD4^+^, Th1, Th2, Th17, and Treg), different phenotypes of CD8^+^ T cells, and various B cell populations, with the exception that the ε subunit appeared to be expressed at somewhat higher levels in cells of the adaptive immune system versus that in NK cells and monocytes (Figure 4, Supplemental Table S1). Again, the expression pattern suggested that some GABA_A_-R heteropentamers are benzodiazepine sensitive, consistent with our findings that murine T cells are benzodiazepine sensitive [71]. We are unaware of any functional evidence of GABA_A_-R modulation of B cells, but given that their expression patterns of GABA_A_-R subunits are generally similar to that of T cells, they may be sensitive to GABA_A_-R agonist modulation. In contrast to the relatively high level of ρ_2_ transcripts detected in human APCs and B cells, human T cells express only low levels of the ρ_2_ subunit. These RNA-Seq gene expression profiles of human immune cells have some overlaps, as well as some contrasts, with the results obtained using RT-qPCR methods [4,6,9,64,72]. Since the RNA-Seq data were obtained using a different technology that is highly quantitative and allows cross-comparison of gene expression levels within a single sample, the presented gene expression profiles provide an alternative resource to help guide hypothesis generation and future research.

Together, these data suggested that many types of immune cells may express GABA_B_-R and GABA_A_-Rs. Systematic studies to identify functional GABA_B_-R and GABA_A_-Rs on immune cells have not been performed, and these receptors may be difficult to detect because: (1) their effects may be subtle, (2) the GABA_A_-R and GABA_B_-R signaling pathways may counter-regulate each other, (3) the effects of GABA-R agonists on other cells in the assay system may mask the effects on cells of interest via bystander effects, and/or (4) the presence of GABA-R subunit transcripts do not always lead to the formation of functional receptors (as in [73]). Further studies, such as electrophysiological studies at the single-cell level, are needed to help resolve whether and which types of immune cells express functional GABA_A_-Rs and GABA_B_-Rs.

The RNA-Seq expression profiles of different types of human immune cells also suggest that immune cells expressed little or no transcripts encoding the classical GABA synthetic enzymes GAD67 (GAD1) and GAD65 (GAD2) (Figure 4, Supplemental Table S1). This may reflect insufficient sensitivity of the RNA-Seq assay since GAD67 and/or GAD65 transcripts were detected in human immune cells by RT-qPCR [9,72] and GAD65 protein was detected in murine APCs via immunoblotting [13]. Interestingly, several different types of immune cells expressed the genes needed to synthesize GABA from putrescine through pathways involving monoamine oxidase B (MAOB), aldehyde dehydrogenase 2 (ALDH2), and ALDH1a1, but not the diamine oxidase (DAO) pathway [60,61,62,63]. These putrescine pathway-related genes were highly expressed in B cells, DCs, and monocytes. Like GABA produced by GAD67, GABA produced from putrescine is released in a nonvesicular fashion through bestrophin (Best1) channels [62,63]. GABA produced through the GAD and/or the putrescine pathways may act in an autocrine fashion as well as having effects on other immune cells in the microenvironment in a paracrine fashion. Finally, all types of immune cells expressed 4-aminobutyrateaminotransferase (ABAT), the enzyme responsible for GABA degradation, as well as some of the GABA transporters (SLC6A11, SLC6A12, and SLC6A13), with especially high levels of SLC6A12 expression detected in DCs, monocytes, and neutrophils.

In summary, we have shown that treatment with lesogaberan, a peripherally restricted GABA_B_-R agonist, displayed the ability to reverse T1D in NOD mice, and to a greater extent when combined with other promising interventive therapies for T1D. The mice that received lesogaberan together with anti-CD3 displayed improved glucose tolerance and had some islets with many insulin^+^ cells, suggesting that GABA_B_-R activation helped to rapidly inhibit β-cell destruction. Finally, our analyses of microarray and RNA-Seq in public databases suggested that GABA_B_-R and GABA_A_-R expression, as well as GABA production, secretion, and uptake may be a more common feature of immune cells than is currently appreciated. While this contention is speculative, the observations provide guidance for future investigations of the roles that GABA_B_-Rs, GABA_A_-Rs, and GABA may play in different types of immune cells.

Limitations of the study: The major limitations of our study include remaining questions as to the molecular mechanisms underlying the action of lesogaberan in restoring euglycemia in diabetic NOD mice, particularly on T cell autoimmunity and pancreatic islet infiltrates in vivo, and the lack of functional validation of gene expression data in different types of immune cells. Additionally, although lesogaberan displayed therapeutic efficacy in the T1D mice studied here, it is unknown whether this will translate to humans given differences in our immune systems and the heterogenicity of T1D in humans.

## Data Availability

The data presented in this study are available on request from the corresponding authors.

## Supplementary Materials

The following are available online at https://www.mdpi.com/2227-9059/9/1/43/s1, Table S1: RNA-Seq numerical data of the expression of genes related to the GABA system.
